# Bortezomib treatment causes long-term testicular dysfunction in young male mice

**DOI:** 10.1186/1476-4598-13-155

**Published:** 2014-06-20

**Authors:** Mi Hou, Emma Eriksson, Konstantin Svechnikov, Kirsi Jahnukainen, Olle Söder, Andreas Meinhardt, Lars Sävendahl

**Affiliations:** 1 Department of Women’s and Children’s Health, Astrid Lindgren Children’s Hospital, Pediatric Endocrinology Unit Q2:08, Karolinska Institutet & University Hospital, SE-171 76 Stockholm, Sweden; 2 Pediatric Hematology and Oncology, Hospital for Children and Adolescen, University of Helsinki, FIN-00290 Helsinki, Finland; 3 Department of Anatomy and Cell Biology, Justus-Liebig-University of Giessen, Aulweg 123, 35385 Giessen, Germany

**Keywords:** Proteasome inhibitor, Bortezomib, Cancer, Mouse, Testis

## Abstract

**Background:**

With increased long-term survivors of childhood cancer patients, therapy-associated infertility has become one of the most common late side-effects and significantly affects their life-quality. Therefore, evaluation of anti-cancer agents on male reproduction and infertility prevention are urgently demanding. The proteasome inhibitor bortezomib has been launched in clinical trials for childhood cancers, however, its potential side effects on reproduction have so far been neither investigated experimentally nor reported in treated children. Thus the present study is designed to explore the impact of bortezomib on male reproductive function and to gain insights into how bortezomib exerts its adverse effects on man gonad, thereby providing pediatric oncologists relevant information.

**Methods:**

35 day-old male mice were treated with one 11-day cycle of bortezomib and then sacrificed 2 days, 45 days, or 6 months later. A mating study was performed in the group followed for 6 months, and their pups were analyzed on postnatal day 50. Serum follicle-stimulating hormone (FSH) and testicular testosterone levels were measured. Testicular morphology was evaluated by light- and electron microscopy, and the underlying mechanisms and pathways of testis damage were investigated.

**Results:**

Testicular damage was visible already 2 days after stopping bortezomib and increased in severity by day 45. Then 80% of seminiferous tubules exhibited hypospermatogenesis with arrest at the levels of spermatogonia, spermatocytes and round spermatids. Germ cells were specifically targeted by bortezomib as evidenced by increased apoptosis mediated through activation of p53 and caspases. Even six months after the bortezomib treatment, testis weight, sperm concentration and seminiferous tubule length remained at a decreased level, indicating that spermatogenesis and tubular outgrowth could not fully recover. Combined with persistently increased serum levels of FSH in these mice, our results demonstrate that bortezomib can have long-term effects on testicular function, although fertility of bortezomib-exposed males remained and their offspring looked healthy.

**Conclusion:**

Bortezomib treatment causes long-term gonadal dysfunction in male mice. Careful monitoring of gonadal function in male childhood cancer patients treated with bortezomib is thus strongly recommended.

## Background

Despite highly improved anti-cancer strategies and increasing numbers of cancer survivors, resistance of malignant cells to chemotherapeutic agents leading to relapsed and refractory cancer remains a major concern. In addition, infertility, one of the severe late side effects of intensive cancer treatment is also an unfavorable factor that negatively influences the quality of life among cancer survivors. Thus, new drugs for efficient treatment of relapse and refractory cancer with fewer side effects on the testis are highly desired.

The 26S proteasome is the enzymatic core engine of the ubiquitin and proteasome dependent proteolytic system (UPS), the major eukaryotic pathway for regulated protein degradation. The UPS plays a pivotal role in cellular protein turnover, protein quality control, antigen processing, signal transduction, cell cycle regulation, cell growth and survival, cell differentiation and apoptosis [1]. Selective degradation of proteins by UPS is a critical determinant for maintaining cellular homeostasis [2], and consequently, inhibition of the proteasome blocks the processes of protein degradation leading to accumulation of proteins that affect multiple signaling cascades within the cells, thereby resulting in cell death and prevention of tumor growth [3].

Bortezomib (Velcade®, formerly known as PS-341, LDP-341 and MLM341) is an inhibitor of the 26S proteasome [4]. Due to its profound anti-tumor effects, bortezomib as the first proteasome inhibitor has entered into the clinic for treatment of adult multiple myeloma [5]. Promising results have been achieved from a phase II clinical trial in adult patients with relapsed or refractory indolent lymphoma [6]. In a recent phase I clinical study in relapsed childhood acute lymphatic leukemia, bortezomib was found to be efficacious when combined with traditional chemotherapeutic drugs [7].

Although the clinical efficacy of bortezomib is evident and many patients tolerate the treatment relatively well, some serious adverse effects, such as neutropenia, thrombocytopenia and heart failure, have been reported [8]. In addition, experimental data have shown that bortezomib selectively targets growth plate chondrocytes, thereby leading to permanent growth failure in young male mice [9]. So far, it is unknown if bortezomib may also impair testicular function and fertility.

During gonadal and germ cell differentiation, ubiquitin, the ubiquitin activating and conjugating enzymes E1, E2, and UBC4, and the ubiquitin C-terminal hydrolase L1 (UCH L1) are all highly expressed by Sertoli cells, spermatogonia, spermatocytes, and spermatids [10]. The UPS is required for the degradation of numerous proteins throughout the mitotic, meiotic and postmeiotic developmental phases of spermatogenesis [11]. The activity of the UPS is high during spermatogenesis [12] due to the demanding requirement of massive breakdown of cytoplasmatic and nuclear proteins during this process [13-15]. Consistent with its multiple functions, alterations of the UPS have been implicated in many pathological processes and sub/infertility. Indeed, it has been observed that targeted disruption of the polyubiquitin gene *Ubb* results in male and female infertility [16] and altered testicular gene expression patterns [17]. Loss of UCHL1, a deubiquitating enzyme responsible for regenerating monoubiquitin from the ubiquitin-protein complex, decreased the rate of apoptosis in the first round of spermatogenesis and increased the numbers of premeiotic germ cells in immature mice [18], whereas asymmetric distribution of UCHL1 in spermatogonia is associated with maintenance and differentiation of spermatogonial stem cells [19]. In contrast, overexpression of UCHL1 was accompanied by reduced PCNA expression and abolishment of apoptosis in spermatogonia, and spermatogenesis was blocked [20]. Moreover, mice lacking the UBC4-testis gene have a delay in postnatal testis development [21]. Based on these reports, we hypothesized that inhibition of the 26S proteasome, the core engine of the UPS would lead to deleterious effects on testicular function. To address this, we designed a study, in which a single cycle of a clinically relevant dose of bortezomib was administered to young male mice at the age of 35 days. Our intent was to evaluate whether bortezomib has testicular effects and impairs fertility of bortezomib-exposed males, and/or has potential impacts on their offspring.

## Results

### Bortezomib reduces testicular weight and sperm concentration

A statistically significant reduction in testicular weight and sperm concentration was observed in all groups treated with bortezomib when analyzed 2 days, 45 days, and 6 months after the last injection (Table 1) compared to controls, whereas body- and seminal vesicle weights were unaffected at all-time points examined (data not shown).

**Table 1:** Testis weight, sperm concentration and length of seminiferous tubule in bortezomib treated and control mice

**Follow-up period**	**Group**	**Testis weight, both sides (mg) n=6**	**Sperm concentration (10**^**6**^**/ml) n = 6**	**Length of seminiferous tubules (m) n = 4**
2 days	Control	91±0.001	3.30 ± 0.543	2.57 ± 0.131
	Bortezomib	83 ± 0.002***	0.89 ± 0.239*	2.36 ± 0.235
45 days	Control	120 ± 0.003	4.62 ± 1.031	2.78 ± 0.103
	Bortezomib	85 ± 0.003***	1.31 ± 0.148***	2.09 ± 0.123*
6 months	Control	114 ± 0.003	11.73 ± 2.027	3.01 ± 0.021
	Bortezomib	80 ± 0.006***	3.12 ± 0.951***	2.40 ± 0.272

Mean ± S.E.M; *P < 0.05, ***P < 0.001.

### Bortezomib causes germinal epithelial damage and decreases longitudinal growth of the pubertal seminiferous tubules

**Figure 1:**
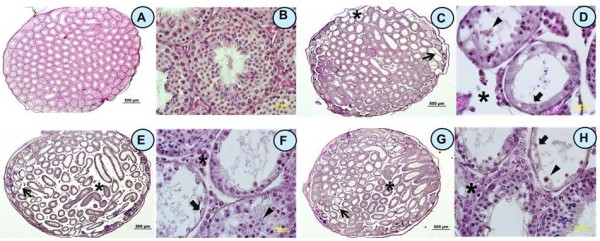
**Effect of bortezomib on testicular histology.** Microphotographs showing testicular histology in a 35 day old untreated control mouse **(A, B)**, and in mice sacrificed 2 days **(C, D)**, 45 days **(E, F)**, or 6 months **(G, H)** after the last bortezomib injection. In bortezomib-treated animals **(C-H)**, spermatogenesis was variably impaired with tubules ranging from Sertoli cells only (arrows) to various degrees of hypospermatogenesis (mixed atrophy) and release of premature germ cells into the lumen (**D**, **F**, **H**, triangles). Loss of germ cells was related to shrinkage of seminiferous epithelium with enlarged interstitial spaces (**C**-**H**, stars). Images were captured at 4× (**A**, **E**, **C**, **G**; bars 500 μm) or 60× (**B**, **F**, **D**, **H**; bars 50 μm) magnification. Corresponding images are displayed side-by-side.

**Figure 2:**
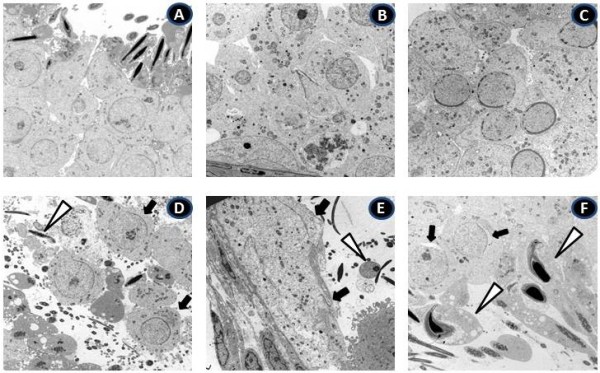
**Electron micrographs of testis pathology associated with bortezomib treatment.** Lower panel shows electron micrographs of testicular sections of mice sacrificed 2 days **(D)**, 45 days **(E)**, and 6 months **(F)** after the last bortezomib injection. Upper panels **(A-C)** display the respective untreated controls. Magnification of all micrographs is 3000×. Damage includes the premature release of round spermatids (**D**, arrows) and elongated spermatids (open triangle) as here illustrated in mice sacrificed 2 days after the last bortezomib injection. In mice sacrificed 45 days post-treatment **(E)**, the germ cell loss had reduced the height of the seminiferous epithelium into a single cell layer (arrows) with malformed nuclei (open triangle). In mice killed 6 months after the last bortezomib injection **(F)**, vacuolization of elongated spermatids (open triangles), impairment of the acrosome formation (arrows), and degeneration of germ cells were evident.

Examination of testicular sections from mice killed 2 days after the last bortezomib injection revealed that approximately 30% of seminiferous tubules were damaged in 4 out of 5 mice examined. The majority of affected seminiferous tubules were located peripherally under the testicular capsule (Figure 1C and D). Impaired seminiferous tubules exhibited hypospermatogenesis with arrest (mixed atrophy) showing spermatogonia (5.8%), spermatocyte (8.7%), and round spermatid (16.6%) as the most advanced germ cell types in seminiferous tubule cross-sections examined, respectively. Only 54.4% of tubules displayed normal spermatogenesis. Altogether 15% of tubules showed a Sertoli cell only phenotype (SCO, Figure 1C and D, arrows), no morphological abnormalities of Sertoli and Leydig cells (LCs) were observed. The length of the seminiferous tubules was comparable to control (Table 1). Severely impaired seminiferous epithelia with degenerating germ cells exfoliating in the lumen of seminiferous tubules were observed under electron microscopy (Figure 2D arrows).

Forty-five days after treatment, 80% of the cross-sections of the seminiferous epithelium revealed a histopathological pattern of damage in all examined animals receiving bortezomib (n = 5, Figure 1E and F). SCO tubules were seen in 19.2% (Figure 1E and F, arrows) with tubules showing spermatogonia (9.6%), spermatocyte (20.1%) and round spermatid (30.7%) as the most advanced germ cell type accounted in the tubules examined, respectively. Only 19.2% of the examined tubules showed normal spermatogenesis. The length of seminiferous tubules in these mice was significantly decreased compared to the controls (Table 1). Electron microscopic examination of testicular sections of these mice revealed that germ cell loss had reduced the seminiferous epithelium to only one single basal cell layer (Figure 2E arrows). Remaining cells showed signs of degeneration, i.e. irregular nuclear outline.

Six months after the cessation of bortezomib treatment, 10-15% of seminiferous tubule cross-sections (mainly located subcapsularly) exhibited hypospermatogenesis with arrest at the level of round spermatids in 2 out of 4 mice examined. One mouse displayed 40-50% of seminiferous epithelial cross-sections with the same pattern of damage (Figure 1G and H, arrows). Compared to the follow-up after 45 days, at 6months the number of seminiferous tubules with normal spermatogenesis had increased from 19.2% to 55.4%, whereas SCO tubules had declined from 19.2% to 9.5%. The percentage of seminiferous tubules that showed spermatogonia, spermatocyte and spermatids as the most advanced germ cell type in this group were 4.1%, 22.9% and 8.1%, respectively. The length of seminiferous tubules increased from 2.09 ± 0.12 m to 2.40 ± 0.27 m but was still shorter than those in the control group (3.01 ± 0.02 m, Table 1) indicating that spermatogenesis and pubertal longitudinal growth of seminiferous tubule were only partially recovered. Electron microscopy showed numerous vacuoles in the cytoplasm of elongated spermatids and electron-lucent areas around the nuclei suggesting fluid accumulation (Figure 2F, open triangles). Acrosome formation was impaired in round spermatids (Figure 2F arrows).

### Bortezomib treatment does not impair fertility

To determine whether a single cycle of bortezomib administration impairs fertility, a mating study was performed 6 months later. This showed that 31% (5 out of 16) of females mated with males who had been exposed to bortezomib became pregnant while 35% (7 out of 20) of those females mated with unexposed males became pregnant (P = 0.86). The litter sizes in corresponding mother groups were 5.0 and 7.3 pups/mother (P = 0.07), respectively. This shows that despite decreased adult testicular volume and sperm counts, the fertility of bortezomib exposed males was not impaired.

### Bortezomib treatment does not affect the first generation

Next, we examined whether pups of bortezomib exposed males were affected. In total, 6 bortezomib-derived male pups were alive and developed normally. Mean testis weight, sperm concentration and body weight in these mice were 86 ± 0.011 mg, 2.60 × 10^6^ ± 0.276/ml and 23.6 ± 0.58 g, respectively, and the corresponding parameters in control pups (n = 13) were 116 ± 0.031 mg, 2.96 × 10^6^ ± 0.465/ml and 23.0 ± 0.36 g, respectively. Frequency of pups surviving until the time of weaning was 48% in bortezomib-derived litters and 57% in control litters. Live-birth index and sex ratio were 13/25 (living offspring/offspring born = 52%) and 13/12(♂/♀) in the bortezomib treated group, while 31/51 (61%) and 25/26 in controls, respectively. No statistically significant difference was detected in any of these parameters. No marked morphological alteration was observed when testicular histology was analyzed under light- and electron microscope after pups in both groups had reached adult age (data not shown).

### Bortezomib increases serum FSH levels

**Figure 3:**
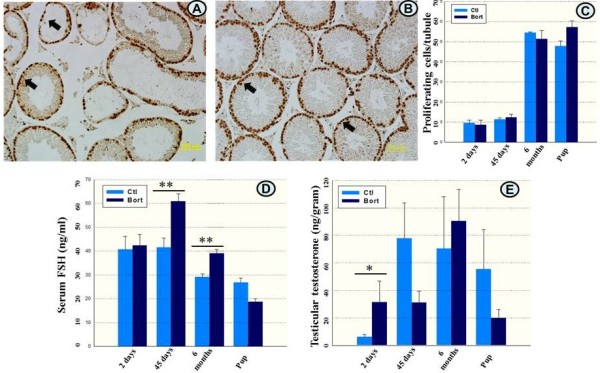
**Immunostaining of testicular sections with PCNA and quantification of proliferating germ cells and levels of serum FSH and testicular testosterone.** Proliferating germ cells in testicular sections from a mouse exposed to bortezomib 45 days earlier **(A)** and a corresponding control **(B)** labeled with brown color (arrows) after immunostaining with PCNA antibody. Quantification of proliferating germ cells per seminiferous tubule in all treated and pup groups, and their respective controls **(C)**. Lower panels show serum FSH **(D)** and testicular testosterone **(E)** levels in 2 days, 45 days, and 6 months follow-up mice and pups of bortezomib treated animals and their corresponding controls. Magnification in **(A)** and **(B)** is 20×. Ctl: control, Bort: bortezomib. PCNA: proliferating cell nuclear antigen.

Serum FSH levels were found to be significantly increased, both 45 days and 6 months after the last injection of bortezomib (Figure 3D). The levels of testicular testosterone were comparable to controls. When assessed 2 days after the cessation of bortezomib treatment, testicular testosterone levels were increased (Figure 3E).

### Bortezomib treatment did not impair spermatogonial proliferation

To assess whether germ cell proliferation was impaired after bortezomib treatment, testicular sections were immunostained applying an antibody against proliferating cellular nuclear antigen (PCNA). No differences in the numbers of proliferative germ cells per seminiferous tubule were found between testes from bortezomib treated and control mice or between the pup groups (Figure 3A, B arrows and C), indicating that bortezomib did not interfere with spermatogonial proliferation.

### Bortezomib induces germ cell apoptosis through p53 and the caspase 8- and 3 pathways

**Figure 4:**
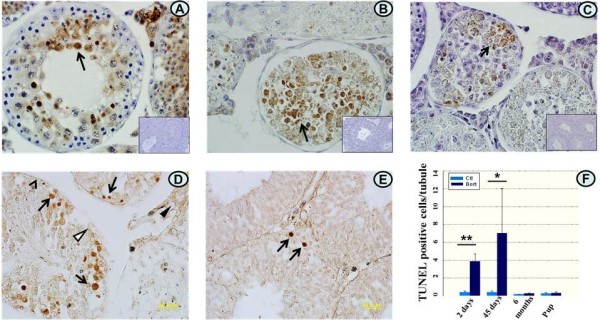
**Immunostaining for p53, active and precursor caspase 8 and active caspase 3, and fragmented DNA.** Upper panels show protein expression of p53 **(A)**, active and precursor caspase 8 **(B)** and active caspase 3 **(C)** as detected by immunostaining (arrows) in testicular sections of mice sacrificed 2 days after the last bortezomib injection. Inserts show negative controls of corresponding sections of **(A)**, **(B)** and **(C)**. Lower panels show TUNEL staining of testicular sections in mice killed 2 days post-treatment **(D)** and their untreated controls **(E)**. Germ cells were positively stained (**D**, arrows) suggesting DNA fragmentation while Sertoli, peritubular and Leydig cells were all TUNEL negative (open triangle, arrowheads, and solid triangle). Panel **(F)** shows the quantification of the numbers of TUNEL positive germ cells per seminiferous tubule in all treated and pup groups, and the respective controls. Magnification of all pictures is 60×. *P < 0.05, **P < 0.01.

To determine how bortezomib caused testicular damage, TUNEL staining, a method to detect DNA fragmentation in nuclei was performed. Various types of germ cells, including spermatogonia, spermatocytes, and spermatids, were stained positively 2 and 45 days after the last administration of bortezomib (Figure 4D, arrows). The germ cells were TUNEL positive, while Sertoli (open triangle), peritubular (arrowhead), and interstitial cells including Leydig cells (solid triangle) did not show positive staining (Figure 4D). In control mice, only a few germ cells were TUNEL positive (Figure 4E, arrow). The number of TUNEL positive cells per seminiferous tubule was significantly increased in mice treated with bortezomib compared to controls (Figure 4F). Thus, bortezomib specifically targets the germ cells by inducing germ cell apoptosis.Apoptosis can be induced by the intrinsic and extrinsic pathways through up-regulation of p53 and caspase 8, respectively, with subsequent activation of the effector caspase 3. To explore the roles of these pathways in bortezomib-induced germ cell apoptosis, testicular sections of mice killed 2 days after the last injection of bortezomib were immunostained for p53, active and precursor caspase 8, and active caspase 3 protein expressions. In bortezomib-treated mice, numerous spermatogonia, spermatocytes and spermatids were stained positively for these markers (Figure 4A,B and C, arrows), whereas controls were negative (inserts, downright corners). This indicates that apoptosis is induced through p53 as well as through the caspase 8- and caspase 3 activating cascades.

## Discussion

A significant acute decrease in the testis weight and sperm concentration was detected 2 days after the last injection of bortezomib. Thirty percent of seminiferous tubules exhibited hypospermatogenesis with arrest at the levels of spermatogonia, spermatocytes or round spermatids. Moreover, 15% of seminiferous tubule displayed SCO. These observations indicate that bortezomib not only affects rapidly dividing germ cells but also decreases the number of quiescent spermatogonial stem cells. Thus, bortezomib is a potent gonadal toxic drug.

The damage to spermatogenetic epithelium increased when the follow-up period was extended to 45 days after bortezomib exposure. At this point, 80% seminiferous tubules exhibited hypospermatogenesis, with spermatogonia, spermatocytes and round spermatids being the most advanced germ cell type. SCO was detected nearly in 20% of examined tubules and serum FSH was elevated. The length of seminiferous tubule was significantly shorter than that in the controls. This means that bortezomib affects also the longitudinal outgrowth of seminiferous tubules in these pubertal mice and exhibits its adverse effects not only on germ cells but also on Sertoli cells. In fact, bortezomib-induced SC toxicity was further confirmed by its action killing spermatocytes and spermatids in the adlumenal compartment, which is normally protected by the blood – testis barrier created by junctions between neighboring SCs [22].

Testis weight, sperm concentration and length of the seminiferous tubule remained at a decreased level six months after bortezomib treatment, indicating that the spermatogenesis and tubular outgrowth was able to only partially recover and that bortezomib can have long-term effects on testicular function.

In the present mouse study, serum FSH levels were increased 45 days after the cessation of bortezomib treatment and these levels were persistently elevated up to 6 months post-treatment, suggesting long-term Sertoli cell dysfunction. Unfortunately, we could not directly study Sertoli cell function by measuring inhibin levels due to limited volumes of serum available. Significant elevated levels of testicular testosterone were also detected in mice 2 days after the last injection. We found that the level of StAR protein (steroidogenic acute regulatory protein) was higher in mice exposed to bortezomib than that in control mice (data not shown). The StAR is an important regulatory protein of steroidogenesis, and administration of bortezomib inhibits the proteasomal degradation of this protein, potentially leading to an accumulation of StAR, thereby enhancing testosterone production. Given the observation that no morphologic alterations of LCs were found and that the levels of testosterone returned to control values after 6 months, we conclude that the steroidogenic function might be unaffected after bortezomib treatment.

The seminiferous epithelium often recovers and rapidly reestablishes normal spermatogenesis through increased germ cell proliferation after toxicant withdrawal [23]. Interestingly, we did not find any such recovery taking place in the seminiferous tubules of those mice being earlier exposed to bortezomib. It is likely that bortezomib blocks the activity of the proteasome which prevents degradation of the cyclin-dependent kinase inhibitors p27Kip1 and p21Cip1, both known to arrest cell cycle progression at G1 [24], and thereby germ cell proliferation.

We can report that bortezomib caused testicular damage by triggering apoptosis in germ cells. Our results further showed that p53 induction and activation of caspases 8 and 3 play important roles in bortezomib induced germ cell apoptosis.

To evaluate whether bortezomib impairs fertility and affects offspring, we performed a mating study in males, which had been exposed to bortezomib 6months earlier. At this point, sperms are differentiated from spermatogonia that were earlier exposed to bortezomib. Fertility of these mice was preserved. There was no difference in the litter sizes and growth of pups was comparable to the controls. No statistical significant differences between pup groups were detected in testicular weights or sperm concentration after pups had reached sexual maturity. Taking together, our data demonstrate that bortezomib treatment does not impair fertility of males, and their offspring are unaffected.

Recently, Manku and colleagues demonstrated that bortezomib blocks the ability of retinoic acid to increase the expression of differentiating spermatogonial markers *Stra8* and *Dazl* in gonocytes isolated from testes of postnatal day 3 rats [25]. Differentiation of neonatal testicular gonacytes to spermatogonia is a critical step for the establishing of the spermatogonial stem cell population, a crucial point for future fertility. In humans, this process lasts from birth up to 4–5 years of age [26], during which period, leukemia, the most common malignant disease in children, occurs. Combining Manku’s finding with our data, we speculate that treatment of prepubertal boys with bortezomib could have deleterious consequences on male reproduction. Further studies to address this issue are demanded.

The bortezomib dose here used (1 mg/kg body weight) has previously been shown to cause 50-80% proteasome inhibition, which is clinically relevant [9,27]. It is also important to point out that in the present study, the impact of only a single cycle of bortezomib was evaluated. In the clinical setting, up to 9 cycles, either with a single drug or in combination with other chemotherapeutic agents is usually used. Higher cumulative doses may significantly affect spermatogenetic recovery leading to sub/infertility.

## Conclusion

The systemic administration of a single cycle of bortezomib was shown to significantly affect spermatogenesis but also to cause Sertoli cell dysfunction. This damage was not fully recovered 6 months after the last administration of bortezomib and led to decreased sperm concentration and adult testicular volume. Based on our experimental data, careful monitoring of gonadal function is suggested in patients being treated with bortezomib.

## Materials and methods

### Animals and bortezomib treatment

**Figure 5:**
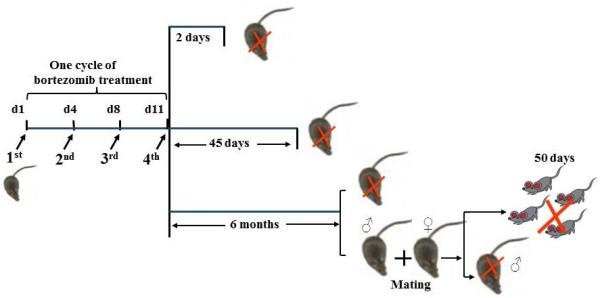
**Protocol for bortezomib treatment and follow-up.** The chart illustrates male mice who at 35 days of age were treated with one 11-day-cycle of bortezomib (1 mg/kg, i.p; injection days indicated: d1, d4, d8 and d11) and then followed for 2 days, 45 days, or 6 months. For mating study, male mice at the same age were treated with bortezomib or vehicle as mentioned above. Six months after these mice and their controls were mated with 8–9 week-old healthy females and killed one day later. The resulting pups were sacrificed on postnatal day 50.

Thirty-five day old (35d-old) C57B young male mice were used (B&K Universal, Sollentuna, Sweden). At this age, the testis development corresponds to what is seen in pubertal boys. The mice were randomized into treatment and control groups, 6 animals per group. In order to mimic the clinical situation, each mouse was injected intraperitoneally (i.p) with a relevant dose of bortezomib [9] (1.0 mg/kg of body weight, dissolved in 0.9% NaCl. Millennium Pharmaceuticals, Inc., MA, Cambridge, UK) while controls received 0.9% of NaCl (vehicle) in intervals as illustrated in Figure 5. To evaluate if bortezomib may cause acute testis damage, what would happen in the testis after it had gone through one cycle of spermatogenesis, and whether potential recovery of spermatogenesis would occur after long-time follow-up, we sacrificed mice 2, 45 days or 6 months after the last injection (Figure 5), respectively. Vehicle-treated mice were pair-fed with equal amounts of food as bortezomib-treated mice up to day 20 in order to avoid any influence of nutritional status. Blood was collected from each animal at the time of killing by heart puncture. Body, testes, epididymidis and seminal vesicles were removed and scaled. One testis from each mouse was fixed in Bouin’s fixative for morphological studies and calculation of length of seminiferous tubule, while part of the other testis was snap frozen and stored at −80°C for testosterone measurement, and rest of the tissue was fixed in glutaraldehyde in an s-collidine buffer for electron microscopic analysis. Use and handling of animals was approved by Stockholm North Animal Ethics Committee (Permits number: N49/06, N9/07 and N283/07).

### Counting the number of sperms

Dual caudate epididymis were dissected out from the epididymides of each animal and placed into 12-well Petri dishes. Each cauda was further cut into 4 pieces with iris scissors and incubated in 1 ml of pre-warmed MEMα medium (Invitrogen AB, USA) in a 37°C water bath for 15 minutes allowing sperms to swim out of the tissues [28]. The number of living sperms in bortezomib- and vehicle-treated mice was calculated after Trypan blue staining under a light microscope.

### Mating study

For mating study, in a second experiment, ten 35d-old mice in each group were treated either with bortezomib or 0.9% of NaCl as stated above and illustrated in Figure 5. Two out of ten mice exposed to bortezomib died and were excluded from the study. Six months after the last injection of bortezomib or vehicle, each male mouse from respective groups (n = 8 and 10 respectively) was caged overnight together with two 8–9 week-old healthy females prior to killing (Figure 5). Fertility index of females (number of pregnant females vs. number of females mated), litter size (mean number of pups/mother), live-birth index (number of live offspring vs. number of offspring produced), sex ratio, frequency of pups surviving until the time of weaning and the nose-tail length were measured and recorded. On postnatal day 50, pups were sacrificed, testis weighed, sperm concentration calculated, and testicular histology evaluated (Figure 5).

### Histological examination

The procedures for fixation and sectioning of testes followed by PAS staining of the testicular sections were conducted as described earlier [29]. Testicular histology was examined from each animal under the light microscope and compared with controls. To estimate seminiferous epithelial damage in detail, more than 100 images were captured serially from testicular sections of each mouse. Between 18–20 round shaped cross sections of tubules were selected randomly from images mentioned above, and at least 4–5 mice per group were analyzed. The relative number of seminiferous tubular cross sections containing Sertoli cell only (SCO) or spermatogonia, spermatocytes, round spermatids and elongating/sperm as the most advanced germ cell type were recorded and presented as mean values ± S.E.M as whole group. For electron microscopy, testicular tissues were fixed and analyzed in the same manner as described previously [30].

### Calculation of the length of seminiferous tubules

In order to measure the length of seminiferous tubules, the diameters of 18–20 round cross-cords randomly selected from each mouse were measured. The proportion of interstitial area was determined by point-counting methodology [31] and seminiferous tubules area was converted by whole testis area (referred to as 100%) subtracting the proportion of interstitial area. Testicular length in each animal was calculated using the formula [32]:

Length of the tubule (m) =

$$\frac{\mathrm{Volume}\;\mathrm{of}\;\mathrm{the}\;\mathrm{testis}\;\left(\mathrm{ml}\right)\times \mathrm{cord}/\mathrm{tubule}`s\;\mathrm{area}\left(\text{\%}\right)}{\pi \times {\left(\mathrm{diameter}\;\mathrm{of}\;\mathrm{the}\;\mathrm{cord}/\mathrm{tubule}\;\left(\mathrm{\mu m}\right)/2\right)}^{2}}$$

and expressed as mean values ± S.E.M as whole group (Table 1).

### Immunohistochemistry

Antigen retrieval was performed in citrate buffer (pH 6.0) containing 0.01% Tween-20 at 95^0^ C for 20 min in a microwave. Testicular sections were incubated with 10% of normal goat serum (for all antibodies) and 3% H_2_O_2_ at room temperature (RT) for 15 minutes, respectively. Thereafter, rabbit anti-mouse p53, active and precursor caspase 8 (Santa-Cruz, sc-28206, sc-7890 respectively, California, USA) and active caspase 3 (Ab-cam, ab-2302, Cambridge, UK) antibodies were all at a 1:50 dilution applied overnight at 4°C. Next day, sections were incubated with a secondary goat anti-rabbit biotinylated antibody at a 1:300 dilution for 1 h at RT followed by incubation with ABC agents and DAB substrate (all purchased from Vector Laboratories, BA-1000, Burlingame, USA), as described earlier [29].

### In situ apoptosis and cell proliferation

TUNEL positive cells in testicular sections were detected by terminal deoxyribonucleotidyl transferase-mediated dUTP nick end labeling as described earlier [30]. Cell proliferation was detected by staining of testicular sections with a rabbit anti-mouse proliferating cell nuclear antigen (PCNA) antibody (1:50, Santa Cruz, sc-7907) followed by incubation with a secondary antibody, ABC agents and DAB substrate as described above. For calculation of TUNEL positive and proliferating germ cells, at least 50 round shaped seminiferous tubule cross-sections from testicular sections of each mouse (n = 4) were counted. The numbers of TUNEL positive and proliferating cells were expressed as positive cells per seminiferous tubule.

### Measurement of serum FSH and testicular testosterone

FSH levels were measured in serum with a rat FSH ELISA kit (Biocode-Hycel, France, Cat: AE R004) according to the manufacturer’s instructions. The concentration of serum FSH was expressed in ng/ml. Intratesticular testosterone concentrations were assayed as described earlier [33]. Briefly, testicular tissues (30–50 mg) obtained from individual mice were homogenized by sonication (2 × 20 sec.) in a sodium phosphate buffer and then centrifuged at 10.000 × g for 10 min. Testosterone concentrations in the supernatants were determined employing the coat-a-count RIA kit (Diagnostic products Corp., Los Angeles, GA, USA) according to the manufacturer’s instructions and expressed as ng/gram tissue.

### Statistical analysis

Results in Table 1 are presented as mean values ± SEM. Differences between two groups were tested by *t*-test followed by a Mann–Whitney Rank Sum Test if the normality test failed. Differences were considered statistically significant for p-values <0.05.

## Abbreviations

PAS staining: Periodic acid–Schiff staining; MEMα medium: Minimal essential medium; SC: Sertoli cell; SCO: Sertoli cell only tube; LC: Leydig cell; FSH: Follicle-stimulating hormone; TUNEL: Terminal deoxynucleotidyl transferase dUTP nick end labeling; DAB: 3, 3’-diaminobenzidine; UPS: Ubiquitin and proteasome dependent proteolytic system.

## Competing interests

The authors declare that they have no competing interests.

## Authors’ contributions

MH: Writing manuscript. MH, EE, AM, LS: Conception and design. MH, KJ, AM: Analysis and interpretation of data (statistical and histological analysis). EE, OS, KJ, KS, AM, LS: Review and/revision of the manuscript. AM, KS: Technical and/material support. OS, AM, LS: Study supervision. All authors read and approved the final manuscript.
